# The anatomy, neurophysiology, and cellular mechanisms of intradental sensation

**DOI:** 10.3389/fpain.2024.1376564

**Published:** 2024-03-25

**Authors:** Elizabeth A. Ronan, Maximilian Nagel, Joshua J. Emrick

**Affiliations:** ^1^ Department of Biologic and Materials Sciences & Prosthodontics, School of Dentistry, University of Michigan, Ann Arbor, MI, United States; ^2^ Sensory Cells and Circuits Section, National Center for Complementary and Integrative Health, Bethesda, MD, United States

**Keywords:** dental innervation, sensory nerve endings, trigeminal somatosensation, tooth pain, teeth, dental pulp

## Abstract

Somatosensory innervation of the oral cavity enables the detection of a range of environmental stimuli including minute and noxious mechanical forces. The trigeminal sensory neurons underlie sensation originating from the tooth. Prior work has provided important physiological and molecular characterization of dental pulp sensory innervation. Clinical dental experiences have informed our conception of the consequence of activating these neurons. However, the biological role of sensory innervation within the tooth is yet to be defined. Recent transcriptomic data, combined with mouse genetic tools, have the capacity to provide important cell-type resolution for the physiological and behavioral function of pulp-innervating sensory neurons. Importantly, these tools can be applied to determine the neuronal origin of acute dental pain that coincides with tooth damage as well as pain stemming from tissue inflammation (i.e., pulpitis) toward developing treatment strategies aimed at relieving these distinct forms of pain.

## 1 Introduction

Teeth have served mammals across their evolution, appearing in their fossil record as early as 140 million years ago (1–3). Most modern species of mammals have retained teeth to facilitate survival through the acquisition and processing of food (mastication) as well as for hunting and defense (1, 4, 5). While specific tooth morphology varies considerably across species, external hard enamel and dentin, along with dense internal sensory innervation, are common anatomical features (5).

Sensory innervation via the somatosensory nervous system enables us to perceive our external world by detecting environmental stimuli. Initial neuronal signals are generated at the peripheral terminals of primary somatosensory neurons (6, 7). These neurons transmit information encoded in neural patterns of impulses from the periphery to the central nervous system. Peripheral somatosensory innervation of the oral cavity, including the teeth, originates from sensory neurons with somas located in the trigeminal ganglia at the base of the skull (Note: sensory neurons in the dorsal root ganglia innervate the neck, trunk, and extremities).

Research over the past half-century has utilized neuroscience techniques to explore the role of tooth-pulp- and dentin-innervating sensory neurons (hereafter referred to as *intradental neurons*) in oral health and disease. This review will delve into the current understanding of intradental innervation, drawing from historical studies, insights from recent single-cell sequencing and transcriptomic methods, and proposed theories of intradental sensory transduction. To begin, we will summarize the tooth anatomy including our current understanding of the intradental sensory apparatus.

## 2 Tooth anatomy and intradental innervation

**Figure 1:**
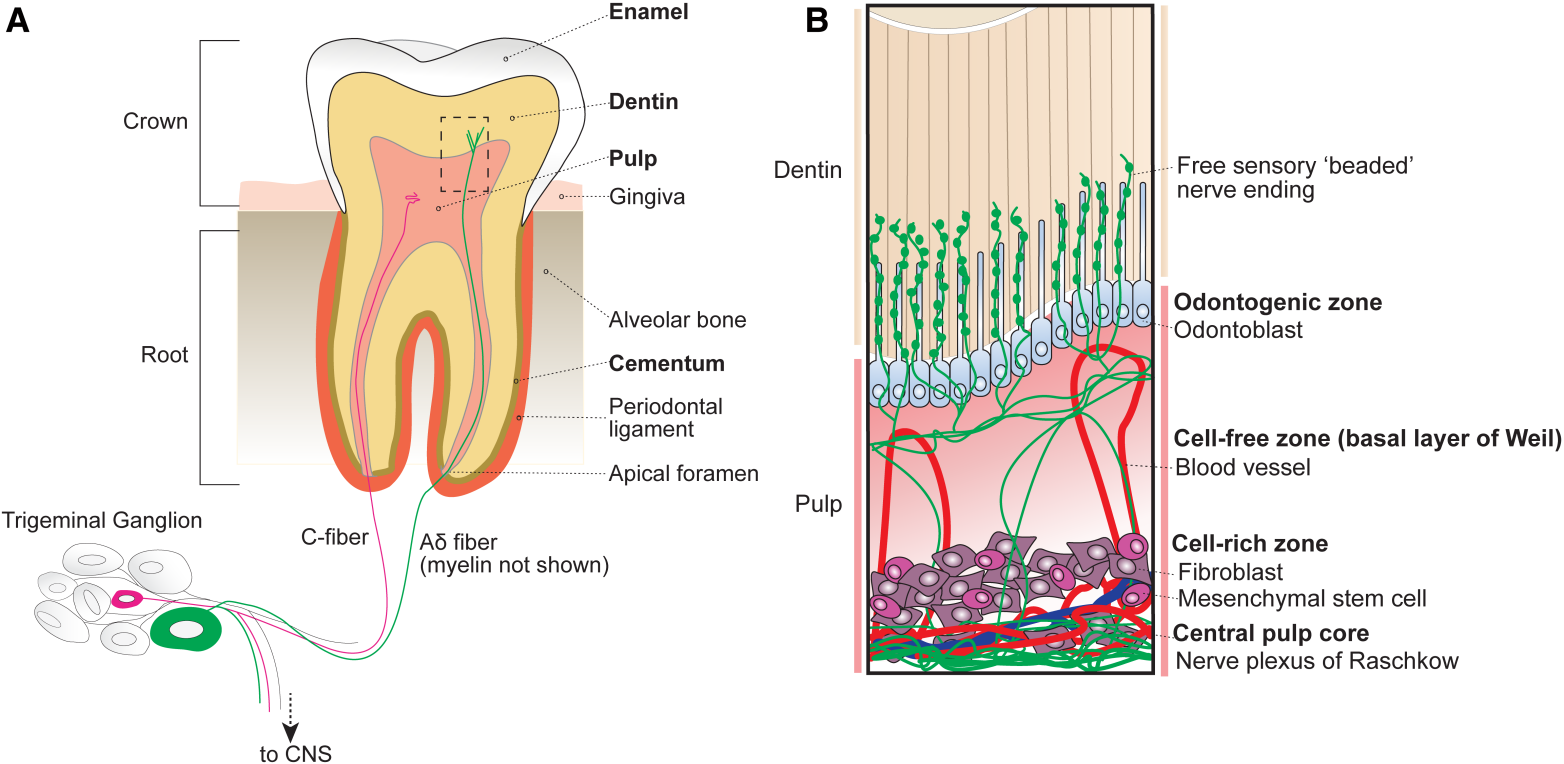
Anatomy of the tooth organ. (**A**) The tooth receives sensory innervation from peripheral sensory neurons whose cell bodies reside in the trigeminal ganglion at the base of the skull. Sensory afferents as well as blood vessels enter the tooth structure at the root apices via small openings known as apical foramen. Surrounding the pulp are several layers of mineralized dentin that support the tooth structure by dissipating external forces. After entering the root, sensory neurons either terminate within the central pulp of the tooth or extend into the inner dentin. The outermost portion of the exposed tooth surface (crown) is covered by protective enamel, which is the hardest substance produced in the human body. The dentin of the tooth roots is encapsulated by mineralized cementum that anchors the tooth within the alveolar bone via the periodontal ligament. Unlike the dentin and cementum layers that can be regenerated across life, enamel is finite. (**B**) Inset from box shown in A depicting the organization of cellular components within the tubular dentin and pulp. The outermost layer of the pulp contains the odontogenic zone, which is comprised of specialized dentin-maintaining polarized cells known as odontoblasts that extend processes into the overlying fluid-filled dentinal tubules. Free sensory endings radiate outwards from nerve bundles at the central pulp core (plexus of Raschkow), towards the occlusal pulp-dentin interface, with some endings extending at or beyond adjacent odontoblast processes. Tubular free nerve endings contain “beaded” swellings filled with synaptic machinery suggesting release of local signals, although their function is not understood. Beneath the pulpal odontogenic zone lies a cell-free zone (also known as the basal layer of Weil), which is largely absent of cellular components. The cell-free zone separates the odontogenic zone from the more central cell-rich zone. Here, mesenchymal stem cells function to replenish odontoblasts as well as local fibroblasts that function to form connective tissues within the pulp. Within the cell-rich zone lies the innermost central pulp core, where the nerve plexus of Raschkow as well as majority of pulp vasculature can be found.

The tooth consists of four major tissues organized into layers (Figure 1A): (1) a central *pulp* that houses living cellular components including vasculature and dense nerve networks, (2) partially mineralized, porous *dentin* comprising the bulk of the crown and root tooth structure that contains nerve endings, (3) a hard mineralized *enamel* cap that protects the superficial crown and (4) a mineralized *cementum* layer that covers the root tooth surface and serves as an attachment to the surrounding alveolar bone via the periodontal ligament.

### 2.1 Pulp

At the innermost part of the tooth lies the dental pulp, which houses living cellular components and unmineralized connective tissues. Based on cellular composition, the dental pulp is divided into 4 main zones from superficial to deep (Figure 1B): (1) odontogenic zone, (2) cell-free zone (basal layer of Weil), (3) cell-rich zone, and (4) central pulp core (8).

The peripheral odontogenic zone consists of specialized cells, odontoblasts, forming a barrier at the pulp-dentin interface. Odontoblasts actively secrete type I collagen and mineralizing agents to form and generate the overlying dentin (9, 10). Intriguingly, odontoblasts also extend a cellular process into the overlying dentin (11, 12). Below the odontogenic zone is the cell-free zone (also known as the basal layer of Weil), which is acellular aside from vasculature and free nerve endings (13). Beneath the cell-free zone lies the cell-rich zone, which houses the majority of pulpal cells including mesenchymal stem cells that replenish surrounding local fibroblasts, peripheral odontoblasts, and locally produced immune cells (14). At the centermost region of the pulp lies the pulp core, which is largely composed of fibroblasts, hosts most of the vasculature that maintains the pulp tissue, and the nerve plexus (8).

**Figure 2:**
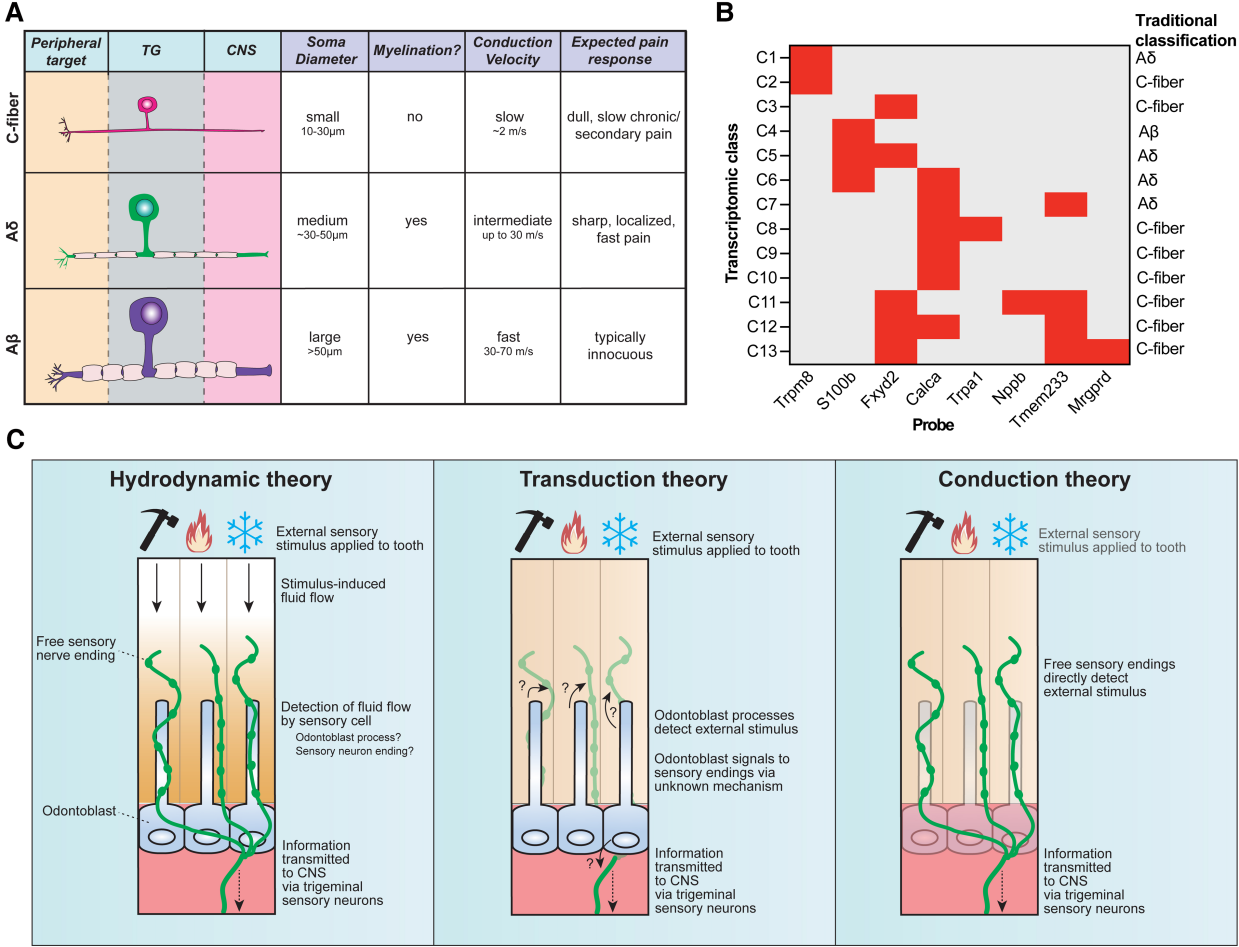
Sensory neuron diversity and proposed mechanisms of intradental sensitivity. (**A**) The traditional classification scheme of somatosensory neurons categorizes trigeminal sensory neurons into three main groups: c-fibers (small somal size, unmyelinated axons, and slow conduction velocity), Aδ (medium somal size, lightly myelinated axons, and intermediate conduction velocity), and Aβ (large somal size, myelinated axons, and fast conduction velocity). Stereotypical pain responses associated with each neuron class are also indicated. (**B**) Recent advances in single-cell RNA sequencing reveal that trigeminal sensory neurons consist of 13 distinct transcriptomic classes. Staining for 8 diagnostic markers via multiplexed in-situ hybridization enables class assignment of trigeminal neurons in tissue sections or whole mount ganglia (66). Graph depicts diagnostic *in situ* probes with corresponding transcriptomic class assignments, as well as how this relates to traditional neuron classes. (**C**) Three major theories have been proposed to underlie intradental sensitivity. (Left) According to the hydrodynamic theory, intradental sensory cells detect fluid movements within the dentin and/or pulp induced by external stimuli to give rise to tooth sensation. Tubular free sensory nerve endings and odontoblast processes are candidate sensory cells to detect stimulus-induced fluid flow. (Center) The transduction theory proposes that odontoblasts, not sensory neurons, function as the primary intradental sensory cells to detect external stimuli. (Right) The conduction theory proposes that tubular free sensory endings directly function as primary detectors of external stimuli. All 3 theories converge on the transduction of trigeminal sensory neurons to relay information to the central nervous system.

The central tooth pulp houses the majority of intradental innervation. Sensory innervation is supplied to the teeth via the superior and inferior alveolar nerve from the maxillary and mandibular divisions of the trigeminal ganglion, respectively (8, 12). Free nerve endings traverse from the center of the pulp into dentin from nerve bundles known as the Raschkow nerve plexus. Historically, sensory neurons have been classified based on morphological and electrophysiological characteristics, broadly dividing them into 3 categories (6): c-fibers (small-diameter, unmyelinated, slow conduction velocity), Aδ (medium-diameter, myelinated, intermediate conduction velocity), or A*β* (large-diameter, myelinated, fast conduction velocity) (Figure 2A). In humans, both large myelinated and small unmyelinated nerve endings enter the tooth root at a small opening known as the apical foramen (12, 15). Consequently, it has long been assumed that intradental sensory neurons include unmyelinated c-fibers, as well as larger, myelinated Aδ and/or Aβ trigeminal sensory neurons.

Tracing of the superior cervical ganglia in large animal models suggests at least a portion of unmyelinated intradental neurons are of sympathetic origin (16). These sympathetic free nerve endings primarily reside along the pulpal blood vessels with highest densities near the pulp horns below the odontoblast layer (17). However, some unmyelinated axons also originate from the trigeminal ganglion, as inferior alveolar nerve degeneration results in a partial reduction of unmyelinated axons at the apical foramen, in addition to complete loss of myelinated intradental neurons (12, 18–20).

Retrograde tracing experiments have shown that in rats, intradental molar neurons are primarily myelinated and medium to large in diameter, indicative of Aδ/Aβ sensory neurons (21, 22). More recently, retrograde tracing revealed that each mouse molar receives unique innervation by a population of approximately 50 large diameter, myelinated neurons (23). Myelinated Aδ/Aβ sensory neurons lose their myelin sheath within the pulp proper before projecting into overlying dentin (24), which may account for prior overestimates of intradental c-fiber contribution. However, the presence of c-fiber conduction velocities in larger animal models suggests that this class may also contribute to intradental sensation (25–28). Whether the lack of evidence supporting intradental c-fibers in rodent models reveals inherent species variability vs. a conserved minor sensory role of c-fibers in tooth sensation remains to be determined.

### 2.2 Dentin

Surrounding the pulp is the dentin, which makes up the bulk of the tooth. Dentin is a layered, mineralized collagenous matrix that provides overall support while effectively cushioning external forces to protect the tooth structure (29). Underlying odontoblasts at the pulp-dentin barrier function to maintain and generate dentin throughout the life of the tooth. Generally, dentin can be divided into several layers based on degrees of hardness and histological composition (10). Intertubular dentin is deeper, most abundant, and features a fluid-filled tubule network that radiates outward from the tooth pulp towards the occlusal tooth surface (5, 30). An outer, less mineralized layer of atubular mantle dentin covers the intertubular dentin. This, in turn, extends to an even more compliant, thin enamel-dentin junction layer (∼25 µm–100 µm thick) that dissipates external forces minimizing the risk and spread of tooth fractures (5, 31).

Within the intertubular dentin, both odontoblasts and myelinated sensory neurons (suggesting Aδ/Aβ-type) extend processes outward from the pulp-dentin border to penetrate approximately one-third of the tubule length (12, 32, 33). This has led to speculation that odontoblasts may function as primary sensory cells that communicate with adjacent free nerve endings to trigger sensation and/or pain (See Section 5 for further discussion on proposed mechanisms of tooth sensitivity). However, electron microscopy studies demonstrate that the dentinal odontoblast process is devoid of pre-synaptic fusion machinery, indicating direct communication within the dentin from the odontoblast process to adjacent free nerve endings is unlikely (12). On the contrary, free nerve endings within the dentin tubules exhibit enlarged swellings along their tips (termed “beaded endings”) that house signaling machinery (including clear and dense core vesicles that express synaptophysin, smooth endoplasmic reticulum, and mitochondria) (8, 12, 34). The function of these beaded endings requires further investigation, but hints at the possibility that tubular free nerve endings could locally communicate with adjacent odontoblast processes.

Anterograde tracing of trigeminal sensory neurons reveals that dentin innervation is not homogenous, with maximal innervation density at the coronal pulp horns and sparse presence in root dentin (12, 33). The receptive fields and branching patterns of individual intradental neurons has been proposed, but not shown. Applying sparse labeling techniques to trace individual neuron branching patterns holds promise to elucidate how intradental sensory neurons individually and might collectively function in the dentin to contribute to tooth sensation.

### 2.3 Enamel

Enamel is the hardest substance in the human body, providing protection to the inner tissues of the tooth organ. Enamel is acellular and composed of highly mineralized hydroxyapatite crystals forming an outermost cap of the tooth crown (30, 35). The enamel layer forms in early tooth development prior to eruption but, unlike dentin, does not regenerate throughout life (36). Despite its hardness, enamel is susceptible to brittle fracture from extreme forces (37, 38) or erosion by bacterial or dietary acids (39, 40). Enamel thickness varies based on species, diet, and tooth type with thicker enamel corresponding to animals who do not regenerate their teeth [e.g., human enamel can be up to 2.5 mm thick at the molar cusp, while mouse enamel is approximately 50–60 μm (41)] (5). Given that enamel is finite and cannot be regenerated, sensory mechanisms safeguarding tooth structural integrity would be presumed to benefit tooth survival and longevity.

### 2.4 Root

The tooth roots are embedded within the alveolar bone anchored by the periodontal ligament. As the anatomical crown is encapsulated by a hard enamel cap, the root dentin is covered by a thin mineralized cementum layer. Despite a comparable mineralization and hardness to dentin, cementum has a lower elastic modulus (5, 42) as it is less prone to impact via direct forces. Unlike enamel, cementum can regenerate via cementoblasts lining the periodontal ligament, allowing self-repair following minor trauma or infection (5, 43, 44). As previously mentioned, apical foramina at the root tips allow for innervation and vasculature to penetrate the tooth interior. Minimal, if any, innervation is observed extending into root dentin and cementum layers (33).

In summary, tooth structure features conserved anatomical specializations including enamel, dentin, and specialized patterns of sensory innervation. Given the tooth's irreplicable nature and essential role in diet and defense, we propose these specializations enable their use as masticatory and defensive tools and contribute to structural protection to preserve the tooth organ.

## 3 Molecular and transcriptomic classification of intradental neurons

The traditional classification of neurons (c-fiber, Aδ, Aβ) has been ubiquitously used in somatosensory neuroscience research for decades (Figure 2A) (6, 45). However, an increasing body of evidence underscores that this system fails to capture the rich molecular heterogeneity that exist within these classes. Work in the last decades has sought to molecularly characterize intradental neurons by evaluating candidate gene expression for various sensory/nociceptive markers, neuropeptides, and ion channels (6). However, establishing a consensus on the molecular expression of intradental neurons and their homogeneity remains challenging due to considerable variability across reports. Discrepancies may be ascribed to differences in model systems (e.g., cultured vs. native intradental neurons, or varying animal models), antibodies/probe variability, and potential bias by evaluating only a panel of candidate molecules.

A substantial body of evidence suggests that at least a portion of intradental neurons exhibit markers for nociceptors. Specifically, multiple reports demonstrate that intradental neurons express calcitonin gene-related peptide (CGRP, *Calca*) (21, 23, 34, 46–49), a neuropeptide that contributes to neurogenic inflammation (50). Furthermore, focus has been placed on evaluating the expression of transient receptor potential (TRP) channels in intradental neurons based on their canonical roles in thermal nociception and/or inflammatory pain (51, 52). Among TRP channels, Trpv1 has been particularly attractive as this thermosensitive channel could represent a potential therapeutic target for pain and inflammation (53, 54). However, while immunohistochemical staining of human coronal dental pulp demonstrates co-expression of TRPV1 in a subset of CGRP+ intradental nerve fibers (55), reported TRPV1 expression in intradental neurons has varied widely (8%–87%) across multiple studies (23, 46, 56–61).

More recent efforts to sequence the transcriptomes of trigeminal sensory neurons have helped refine their classification. Single-cell transcriptomic profiling has identified around a dozen major transcriptomic classes of sensory neurons (62–65) which can be assigned to cells in tissue sections and whole-mount trigeminal ganglia using in-situ hybridization (ISH) staining (Figure 2B) (66). Retrograde labeling of trigeminal sensory neurons innervating mouse molars in conjunction with *in situ* classification has recently allowed the determination of the transcriptomic diversity of neurons innervating molar teeth, revealing each tooth receives contributions from a population of specialized trigeminal neurons that express nociceptive markers as well as genes associated with fast-conducting neurons (*S100b*) reflecting either Aδ or Aβ (23). Intradental neuron expression of *S100b* and *Calca* (encodes CGRP) or *S100b* and nociceptive voltage-gated sodium channel Nav1.8 (*Scn10a*) aligns with previous reports (58, 61), and designates the majority as large diameter, C6 cells (23) which would be traditionally classified as Aδ mechano-nociceptors. Importantly, this study indicated that few intradental neurons likely represent the small-diameter c-type classes (C2, C7-10) that express *Trpv1*, *Trpm8*, and *Trpa1* (23). While this does not rule out that TRP channels may still be present and play a role in intradental neuron physiology, it does fall in line with those previous reports that indicate lower expression levels of these channels. Of note, intradental neurons demonstrated enriched expression of nociceptive marker 5-hydroxytryptamine (serotonin) receptor 3A (Htr3a) and the touch receptor Piezo2 (23). These channels may contribute to intradental neuron physiology and warrant further investigation with tractable genetic models.

Similarly, a subsequent transcriptomic profiling of acutely cultured mouse molar neurons showed the majority feature enriched expression of *S100b*, *Calca*, and *Piezo2* (67)*.* Intriguingly, this study identified additional populations of intradental neurons which showed enriched expression of *Trpv1* and *Trpa1* (67)*.* While these neurons were assigned to C7/C8/C10 classes based on the presence of TRP channels, they also featured high expression of *S100b* associated with myelinated neurons not c-fibers.

## 4 The function of intradental sensory neurons

Given that intradental neurons richly innervate the inner dentin and tooth pulp, this begs the question as to how they contribute to sensation. Patients typically describe conscious tooth sensation as painful, and many studies assume that pain is the only output from intradental neurons (68). Certainly, infections that produce inflammation of the pulp are associated with dramatic inflammatory pain (69, 70). However, considering that pain typically serves a protective role to prevent tissue injury and encourage healing, the utility of inflammatory pain from the tooth is ambiguous since it often requires clinical interventions for resolution in human patients. Nociceptive pain from operative dental procedures results from the removal of tooth structure. Here, accompanying pain serves as a protective signal indicating direct tissue damage. Given that dental drilling represents an artificially amplified stimulus, pain may also represent an exaggerated output of intradental neurons. Consequently, natural factors damaging the tooth might produce different, less severe sensation.

Supporting this notion, psychophysical studies using electrical stimulation on healthy human teeth indicate more diverse perceptual outputs from intradental neurons (71). Lower intensity pulses elicited pre-pain tingling sensations that transition into intense pain when delivered at higher frequencies (8, 68, 71, 72). Pulse intensities that would be predicted to activate myelinated fibers produce pre-pain sensation and also trigger a jaw-opening reflex (73). This indirectly suggests that myelinated intradental neurons likely underlie both pre-pain and pain sensations, and may also initiate protective reflex(es) to preserve the tooth organ. The detection of *Piezo2* in intradental neurons through recent transcriptomic analyses hints that these neurons could detect lower intensity forces, as this ion channel underlies discriminative touch in the skin (8, 23, 67, 72, 74–76). While human patients are capable of sensing innocuous touch vibration applied to the teeth (77), it has not been demonstrated whether intradental vs. surrounding tissue innervation is responsible for vibration sensation.

Single unit recordings of the inferior alveolar nerve of large animal models (primarily cats and dogs conducted in the 1970s and 1980s) support innervation of the dentin by myelinated intradental neurons (25–28, 78, 79). Activation of intradental neurons in large animal models using an electric pulp tester evoked responses from fibers with conduction velocities in the Aδ-range (25–27, 78, 79). Corroborating these findings, studies performed using mechanical probing, cold application, scraping, and air-blast of exposed dentin similarly triggered neuronal responses with conduction velocities in the Aδ range (27, 80–82). While intradental neurons responded in these extreme manipulations where the dentin was exposed, their response profile within the context of intact enamel, behavioral outputs, as well as associated perceptions remain largely unexplored.

Though less consistently reported, a handful of animal studies have found responses with c-fiber conduction velocities to noxious stimuli (temperature, mechanical) and inflammatory mediators applied to teeth (25–28). For instance, mechanical probing of exposed tooth pulp, but not dentin, activates fibers with slower conduction velocities, suggesting distinct pulpal localization of c-fibers (83). Broadly, c-fiber activation is thought to present as persistent, dull pain—similar to inflammatory pain (84, 85). This opens a possibility that activation of Aδ vs. c-fibers may give rise to differing types of intradental sensation. In this model, dentinal Aδ fibers would transduce superficial stimuli, while pulpal c-fibers would respond when stimuli penetrate deeper into the pulp or in the context of pulpal infection. In support of this model for functional divergence, one study using intact cat canine teeth found intense heating induces a biphasic response marked by acute Aδ-range action potentials followed by a prolonged activation of c-fiber spikes (80). Furthermore, application of the inflammatory mediator bradykinin to exposed pulp elicits action potentials from c-fibers (27) as well as dull pain sensations in humans (86). Taken together, this body of work strongly suggests that an elusive subpopulation of intradental neurons represents c-fibers that trigger pulpal pain but lack an obvious function outside of the context of extreme stimulation or infection.

Outside of their direct sensory roles, intradental neurons may also contribute to local tissue responses. The distribution and patterning of free sensory nerve endings within the pulp-dentin complex of a fully developed tooth is not static, but can dynamically remodel in response to injury or infection. CGRP+ sensory endings exhibit branch sprouting surrounding sites of pulp damage, although prolonged and extensive damage leads to eventual pruning and denervation at the damage site (48, 87). Denervation by resection of the inferior alveolar nerve is shown to accelerate necrosis induced by pulp damage (88), suggesting that sensory fibers may contribute to pulp-dentin regeneration in response to injury. Indeed, inflammation-induced CGRP release in the pulp has been shown to regulate capillary blood flow and induce tissue regeneration (89). Further research is necessary to determine if intradental sensory neurons respond to inflammation by releasing CGRP or other neuronal factors in order to promote dentin repair.

## 5 Proposed mechanisms of tooth sensitivity

In most peripheral tissues, free sensory nerve endings are directly activated by sensory stimuli such as mechanical forces or temperature (90). However, the tooth organ is structurally distinct given its encapsulated hard surface, multiple layers of mineralization, and fluid-filled dentin tubules within which the specialized sensory endings and odontoblast processes permeate. This specialized anatomy opens a possibility that sensory detection within the teeth may differ compared to other peripheral targets. Several theories have been proposed regarding tooth sensation: (1) *hydrodynamic theory*—sensory neurons are indirectly activated by external stimuli through induced fluid movement within the tubules, (2) *transduction theory*—odontoblasts act as primary sensory cells signaling to sensory neurons, and (3) *conduction theory*—sensory neuron endings within the inner pulp and dentin tubules are directly activated by sensory stimuli (Figure 2C). It is important to note that these theories are not mutually exclusive and elements of each may function together to give rise to intradental sensation. In this section, we will briefly reflect on current evidence regarding these theories of dental sensitivity.

### 5.1 Hydrodynamic theory

Investigations into the functionality of intradental neurons have largely been guided by the hydrodynamic theory, first proposed by Brannstrom (91). This theory asserts that sensory cells are activated by fluid flow within the dentinal tubules as a consequence of external stimuli. Here, fluid movements are proposed to amplify external stimuli facilitating their detection by inner sensory cells.

Evidence supporting the hydrodynamic theory stems from both clinical observations and *in vivo* functional studies. Both have shown that sensations elicited from direct dentin stimulation are heightened when the dentinal tubules are exposed and thought to increase tubular fluid flow (8, 28, 80, 92–94). Supporting this idea, re-blocking tubules with composite reduces the sensitivity (95). However, the reduced sensitivity of dentin following composite application is confounded by disruption of the native tooth composition and structure. Additionally, these observations do not rule out sensory stimuli are directly activating sensory endings (see below, conduction theory).

Studies have sought to estimate fluid flow through the tubules (in terms of both flow rate and direction) in response to external stimuli. Interestingly, most external stimuli tested induce measurable fluid flow within dentin and pulp (96–99). Several independent reports estimate similar pulpal flow rates (in the range of 3.5–22.2 × 10^3^ picolitres(pl)/second), showing fluid flow rate is most concentrated at the region of stimulation and that most mechanical stimuli give rise to inward (or “pulpward”) flow (96–99). Thermal stimuli may induce bidirectional fluid movement within enamel and/or dentin tubules due to thermal contraction/expansion (100, 101). Measurements estimating induced intradental fluid flow in response to thermal stimulation corroborate computational modeling results that cold stimuli induce outward flow, while heating induces pulpward fluid flow (96, 102, 103). Intradental neurons demonstrate lower response threshold to outward fluid flow based on single unit recordings. Conversely, inward pulpward flow requiring a much higher flow rate to elicit a response (104–106). In support of this idea, human patients report heightened sensitivity in response to outward pressure applied to exposed dentin (107). The mechanism or the utility of this preferential response is not clear. Notably, the hydrodynamic theory predates the cloning and study of thermosensitive ion channels that respond directly to temperature (108, 109) and most of these have yet to be explored functionally, *in vivo* within the context of intradental sensation.

Overall, the hydrodynamic theory posits that fluid flow induces the activation of intradental sensory cells, necessitating the activation of a molecular mechanotransducer. Because external stimuli always impact internal fluid flow, it will remain challenging to determine the physical initiator of intradental sensation. Assaying qualitative differences from multiple modalities of tooth stimulation (hot vs. cold vs. touch) could be informative considering the hydrodynamic theory posits that all would converge on fluid flow. Furthermore, the sensory cell responsible for detecting fluid movement within the tubules is debated, with proposed candidates being tubular odontoblast processes or free nerve endings (transduction vs. conduction theory). *Piezo2* emerges as a potential molecular candidate as it may be expressed in both odontoblasts and intradental neurons, but further investigation *in vivo* is required (66, 67, 110).

### 5.2 Transduction theory

The transduction theory suggests that odontoblasts function as the primary sensory cells that signal to free nerve endings. Odontoblasts could represent excitable cells *in vivo* based on their expression of ion channel receptors and electrophysiological responses in culture (111–122). However, as with any cells, *in vitro* culture may significantly alter the expression pattern and excitability of odontoblasts and may not provide an accurate representation of their native state*.* The reported variability in Trp channel expression by odontoblasts lends further credence to this concern (111). While this has been a more active area of research in recent decades, direct functional evidence is limited due to the technical challenges of assaying native odontoblasts. Recently, TRPC5 in odontoblasts was proposed as the direct cold sensor, contributing to dentinal cold sensitivity (113). Trpc5 expression appears limited to root-adjacent odontoblasts (113), where sensory innervation into dentinal tubules is notably sparse (12, 33). Odontoblast morphology has also been shown to drastically differ in coronal vs. root dentin (11), further suggesting potential functional heterogeneity in odontoblast populations based on anatomical location.

It remains an open question as to how odontoblasts would communicate with sensory neurons. While nerve endings and odontoblast processes are adjacently oriented within a single tubule, electron microscopy studies suggest they do not directly contact or connect via gap junctions (12, 32). Electron microscopy demonstrate tubular odontoblast processes are devoid of axoplasmic organelles or signaling components (12). Thus, if odontoblasts do represent sensory cells, their ability to relay this information to sensory nerves must be non-traditional. One model proposes that, when excited, odontoblasts release an extravesicular signaling molecule to bind receptors on adjacent free nerve endings. Extracellular ATP (eATP) remains a candidate given a body of *in vitro* evidence using co-cultured odontoblasts and trigeminal sensory neurons. P2X_3_ immunostaining has been demonstrated in a subset of human dental pulp afferent fibers in addition to odontoblasts (123). A similar relationship between epithelial cells and sensory neurons has been reported in the skin. Epithelial keratinocytes function as nonneuronal sensory cells that detect touch and temperature to release ATP onto its cognate receptor P2X4 expressed on nearby free sensory endings (124, 125). Further *in vivo* evidence is necessary to demonstrate that intradental sensory neurons are responsive to ATP.

### 5.3 Conduction theory

The conduction theory proposes that free sensory endings directly detect sensory stimuli applied to the tooth, then transduce electrical signals to the nervous system. Indeed, somatosensory neurons express all relevant molecular machinery to detect external stimuli and their heterogeneity and anatomical localizations reflect this specialization. Supporting a model that the neurons initiate signaling within the tooth, dentinal sensory endings have been shown to contain axoplasmic organelles that are reminiscent of vesicles found in presynaptic neurons. Activated sensory neurons may signal to odontoblasts to initiate protective dentin production. Interestingly, a recent study of the inferior alveolar nerve found that chronic constriction injury (CCI), which has been shown to induce chronic neuronal activation (126), gave rise to significant pulp calcification. Further investigation is required to determine whether intradental neurons indeed function as the primary detectors of sensory stimuli as opposed to odontoblasts. Nevertheless, intradental neurons play an indispensable role in transmitting sensory input from the teeth to the central nervous system.

The encapsulated nature of the tooth structure has made it challenging to parse out the extent these three theories contribute to overall tooth sensitivity. Future studies using targeted genetic approaches such as cell-specific ablation in conjunction with functional assays will shed light on the cellular mechanism of intradental sensitivity.

## 6 Concluding remarks

This review presents an overview of tooth structure and innervation toward understanding tooth sensation. The distinctive structure of the tooth organ features elements that are conserved among mammals and lead to the protection of these essential organs. Enamel covers the outer tooth and myelinated sensory neurons extend from the pulp and penetrate the dentin tubules. The tooth pulp is densely innervated by sensory neurons which have been further described based on their transcriptomic profiles. Most intradental innervation originates from myelinated trigeminal neurons that terminate in the inner dentin. Functional studies and clinical observations investigating sensory innervation in both animal models and humans have demonstrated pain is a predictable consequence of extreme scenarios of intradental sensory neuron activation (e.g., when tooth structure is damaged and/or dentin is exposed). The contribution of sensory neurons to sensation in an intact tooth has not been defined.

Future research is needed to determine the cellular and molecular origin of tooth sensation as well as how molecularly-defined intradental neurons respond to a range of tooth stimulations and produce nociceptive vs. inflammatory tooth pain. Given the advances of genetic tractability using murine models in recent decades, mouse studies show promise for elucidating the identity of intradental sensory neurons. However, when interpreting rodent data, it is critical to note that studies on molars, which are structurally similar to human teeth, provide the most relevant comparisons considering these do not regenerate throughout life. Ultimately, these insights will contribute to the development of targeted clinical anesthetics and treatments for dental pain
